# Neurodegenerative Properties of Chronic Pain: Cognitive Decline in Patients with Chronic Pancreatitis

**DOI:** 10.1371/journal.pone.0023363

**Published:** 2011-08-18

**Authors:** Marijtje L. A. Jongsma, Simone A. E. Postma, Pierre Souren, Martijn Arns, Evian Gordon, Kris Vissers, Oliver Wilder-Smith, Clementina M. van Rijn, Harry van Goor

**Affiliations:** 1 Donders Centre for Cognition, Donders Institute for Brain, Cognition and Behaviour, Radboud University Nijmegen, Nijmegen, The Netherlands; 2 Department of Learning and Development, Behavioral Science Institute, Radboud University Nijmegen, Nijmegen, The Netherlands; 3 Research Technical Support Group (RTOG), Department of Social Psychology, Radboud University Nijmegen, Nijmegen, The Netherlands; 4 Research Institute Brainclinics, Nijmegen, The Netherlands; 5 The Brain Resource International Database and the Brain Resource Company, Ultimo, Australia; 6 Pain and Nociception Neuroscience Research Group, Department of Anaesthesiology, Pain and Palliative Medicine, Radboud University Nijmegen Medical Centre, Nijmegen, The Netherlands; 7 Pain and Nociception Neuroscience Research Group, Department of Surgery, Radboud University Nijmegen Medical Centre, Nijmegen, The Netherlands; National Institutes of Health, United States of America

## Abstract

Chronic pain has been associated with impaired cognitive function. We examined cognitive performance in patients with severe chronic pancreatitis pain. We explored the following factors for their contribution to observed cognitive deficits: pain duration, comorbidity (depression, sleep disturbance), use of opioids, and premorbid alcohol abuse. The cognitive profiles of 16 patients with severe pain due to chronic pancreatitis were determined using an extensive neuropsychological test battery. Data from three cognitive domains (psychomotor performance, memory, executive functions) were compared to data from healthy controls matched for age, gender and education. Multivariate multilevel analysis of the data showed decreased test scores in patients with chronic pancreatitis pain in different cognitive domains. Psychomotor performance and executive functions showed the most prominent decline. Interestingly, pain duration appeared to be the strongest predictor for observed cognitive decline. Depressive symptoms, sleep disturbance, opioid use and history of alcohol abuse provided additional explanations for the observed cognitive decline in some of the tests, but to a lesser extent than pain duration. The negative effect of pain duration on cognitive performance is compatible with the theory of neurodegenerative properties of chronic pain. Therefore, early and effective therapeutic interventions might reduce or prevent decline in cognitive performance, thereby improving outcomes and quality of life in these patients.

## Introduction

Chronic pancreatitis is a serious medical disease characterized by inflammation of the pancreas resulting in progressive and irreversible morphological changes and often end-stage exocrine/endocrine failure [1]. Alcohol abuse is the most common etiology in chronic pancreatitis, preceding the disease in 55%–80% of chronic pancreatitis patients in industrialized nations [2]. Severe chronic abdominal pain is the major presenting complaint present in 80%–90% of patients during the course of the disease [3], [4]. Pain can be considered the most important factor causing a substantial loss of quality of life [5]. The intense relapsing or persistent pain in chronic pancreatitis leads to recurrent hospitalizations, multiple medical interventions, opioid addiction [6], [7] and is associated with major socio-economic problems [8]. The pain in chronic pancreatitis is still not completely understood, but does involve peripheral nociceptive, peripheral neuropathic and central neuroplastic mechanisms [9].

It is now well accepted that neuroplasticity, i.e. altered central pain processing, plays an important role in the development of chronic pain [10]. Once pain has become chronic, as in chronic pancreatitis, it is difficult to treat satisfactorily [3]. Thus surgical treatments aiming to interrupt nociceptive input from the pancreas, e.g. celiac plexus blockade, pancreatic denervation, or total pancreatectomy, fail to relieve pain in a substantial proportion of patients with chronic pancreatitis [11]. In general, long-term quality of life remains poor after surgery in patients with chronic pancreatitis [12]. The accompanying invalidity, reduced ability to work, induced sleep disturbances, increased anxiety and depressive symptoms [13], [14], make chronic pancreatitis pain an unsolved healthcare problem in society.

Many patients suffering from chronic pain report cognitive complaints. There is substantial evidence that chronic pain can impair cognitive abilities [15], [16], [17], [18], [19], [20], [21], [22], [23], [24].

However, most of these studies included patients with unspecified pain and pain syndromes of varying etiologies [22], [25]. Moreover, explanations for the observed cognitive decline remain scarce. Possible explanations might be related to the observed chemical and structural changes in the brain of patients suffering from chronic pain [26], [27], [28]. Indeed, MRI research has shown that in patients suffering chronic pain, gray matter density is decreased, especially in the prefrontal cortex and the thalamus [29], [30], [31], [32]. Apkarian and colleagues reported that chronic pain patients were impaired on an emotional decision task, a test that has been directly linked to functional properties of the frontal lobe [33]. The authors explained their findings in terms of loss of gray matter in the frontal lobe of chronic pain patients [33]. To our knowledge, this was the first study directly linking neurodegeneration, chronic pain and a specific cognitive deficit.

In acute and chronic neurodegenerative diseases, neuronal cell death is also an important factor underlying the observed decline in cognitive functions [34]. In view of shared disease mechanisms (e.g. neuronal necrosis), it has been suggested that chronic pain should also be considered a neurodegenerative disorder [26].

Apart from severe pain, many chronic pancreatitis patients report also other factors that have been associated with a decrease in cognitive functions, such as depressive symptoms [35], [36], [37], sleep disturbances [38], use of opioid medication [39], and a history of alcohol abuse [40].

The objectives of this study were 3-fold. Firstly, we wanted to examine whether chronic pancreatitis pain is associated with cognitive decline. A second objective was to examine whether documented cognitive deficits are related to pain duration, supporting the hypothesis that chronic pain is a progressive neurodegenerative disorder. Thirdly, our aim was to examine to what extent other individual factors, e.g. depressive symptoms, sleep disturbance, opioid medication and a history of alcohol abuse, contribute to the cognitive decline of patients with chronic pancreatitis. To this end, the neuropsychological profile of chronic pancreatitis patients was documented by means of a complete neuropsychological test battery and compared with the neuropsychological profile of healthy controls matched for age, gender, and education.

## Methods

### Study design

Over a ten-month period, sixteen patients with confirmed chronic pancreatitis and associated chronic pain were referred to the Brainclinics Research Institute, Nijmegen, the Netherlands, for standardized assessment of their cognitive abilities. The diagnosis of pancreatitis was based on a standard battery of history, laboratory tests and radiological findings. Patients were ambulant and randomly selected from the outpatient clinic. Patients with persistent alcohol use were excluded from the study. The control group consisted of 16 healthy volunteers who were matched to the chronic patient group according to age, gender, and years of education. For all participants, the standardized neuropsychological assessment protocol of the Brain Resource International Database was used [41]. For a description of demographic variables of the healthy volunteers and the chronic pancreatitis patients, see Table 1 upper panel.

**Table 1:** Demographic and individual variables of the participants.

Demographic variables of the participants		Healthy controls	Patients
Number of participants	(n)	16	16
Age (years)	(mean ± SD)	48.0±11.3	49.5±11.9
Male/Female	(n/n)	10/6	10/6
Years of education	(mean ± SD)	11.9±2.9	11.8±3.1

Individual variables of the participants			
Pain duration (years)	(mean ± SD)	0	6.12±3.01
Depression (DASS-21)	(mean ± SD)	1.75±2.38	5.27±4.78
Sleeping disturbance	(yes/no)	7/9	6/10
Opioid medication	(yes/no)	0/16	8/8
History of alcohol abuse	(yes/no)	0/16	8/8

For the healthy volunteers, medical ethical approval to collect the data was obtained (Committee on Research involving Human Subjects, Region Arnhem-Nijmegen nr. 2002/008). The patients were all referred by their physician in charge for neuropsychological testing, as part of their medical follow up. Written informed consent to use the data for scientific purpose was signed by all subjects, both healthy volunteers and patients.

### Data collection

Cognitive abilities were assessed by means of the Integneuro test battery using an automated touch screen tool [41]. The participants were instructed to refrain from smoking and drinking caffeine 2 hours before the study. They were seated in front of a touch screen computer (NEC MultiSync LCD 1530 V) in a sound attenuated room. Task instructions and materials were pre-recorded and delivered in a standardized way via headphones and using the visual display on the touch screen computer. The Integneuro test battery consisted of 13 tests, which covered three clusters of cognitive domains, namely psychomotor performance, memory, and executive functions. Some tests consisted of two or more subtests. For a description of the tests used, see [41] and Appendix S1. The total test battery took approximately 50 minutes to complete.

### Pain duration

The period (in years) that the chronic pancreatitis patients were under medical control for pain treatment was taken as measure of pain duration. Matched controls were free of chronic pain and scored 0 on this variable.

### Additional variables

Apart from group (chronic pancreatitis patients versus matched healthy volunteers) and pain duration, four covariates were included in this study; depressive mood, sleep disturbances, opioid use, and a history of alcohol abuse.

In both patients and healthy volunteers, depressive mood was assessed by the short-form version of the Depression Anxiety Stress Scales (DASS-21) [42]. The DASS-21 is comprised of a 21-item questionnaire referring to the severity/frequency of negative emotional symptoms experienced “over the past week” with each item rated on a 4-point scale. The score for depression was calculated by summing the scores for the relevant items.

Information about sleep disturbance and opioid medication was obtained through a screening questionnaire leading to either the presence of sleep disturbances or not in both the patients and healthy volunteers. Information about a history of alcohol abuse in the past and or the use of opioid medication was extracted from the medical files of the patients (Table 1 lower panel). Sleep disturbance, use of opioid medication, and a history of alcohol abuse were scored in a dichotomous way (yes/no).

### Data and statistical analysis

The data of the current study were considered to be hierarchical, meaning there were measurements (level 1) within participants (level 2) and the test scores from these measurements were clustered in three different cognitive domains: psychomotor performance, memory, and executive functions. From this we expected dependency within the data. Therefore, a multivariate multilevel analysis was applied for the current data [43], [44] The use of a multilevel approach was further supported by its permitting the retention of participants that had missing data amongst the dependent variables (see Table 2).

**Table 2:** Raw scores of the participants of all the variables.

Cluster	Test nr	Variable measured	Healthy controls		Patients	
			Mean (SE)	N	Mean (SE)	N
Psychomotor	1	Tapping freq. (dominant) (#)	164 (9)	16	133 (13)	11
		Tapping freq. (non-dominant) (#)	154 (7)	16	130 (11)	11
		Tapping variability (dominant) (ms)	21.1 (3.7)	16	77.3 (22.9)	11
		Tapping variability (non-dominant) (ms)	29.1 (4.6)	16	67.6 (22.5)	11
	2	Target detection (ms)	321 (12)	13	375 (24)	13
	3	Choice Reaction Time (ms)	688 (19)	16	816 (40)	16
	4	Working Memory Reaction Time (ms)	503 (36)	13	604 (40)	15
Memory	5	Verbal word Learning trials (#)	7.5 (0.3)	16	6.9 (0.4)	16
		Verbal word Learning trials (slope)	1.0 (0.1)	16	1.0 (0.2)	16
		Verbal word Delayed recall (mean)	6.1 (0.6)	16	5.7 (0.6)	16
		Verbal word Recognition (sensitivity)	0.7 (0.3)	16	0.7 (0.4)	16
	6	Maze A (s)	279 (36)	16	353 (56)	14
		Maze B (s)	242 (34)	16	305 (48)	14
		Digit span forward task	5.7 (0.3)	16	5 (0.3)	16
	7	Digit span backward task	3.7 (0.3)	16	3.5 (0.4)	15
	8	Visual Span	6.4 (0.7)	15	6.4 (0.7)	13
Executive	9	Switching of Attention 1 (s)	22.3 (1.3)	16	30.8 (3.4)	16
	10	Switching of Attention 2 (s)	56.3 (5.7)	16	71.9 (7.0)	16
		Switching of Attention 2 (errors)	0.9 (0.3)	16	3.4 (1.1)	14
	11	Verbal Interference (correct)	9.4 (0.9)	16	6.0 (0.9)	16
		Verbal Interference (errors)	1.2 (0.3)	16	1.8 (0.3)	16
	12	Intrusions	0.1 (0.1)	16	0.4 (0.1)	16
	13	Go-NoGo (ms)	311 (15)	13	350 (21)	12

Mean and standard error (SE) with number of participants (N) of the unstandardized cognitive tests scores in the psychomotor -, memory - and executive functioning cluster.

A multivariate multilevel analysis with fixed occasion models was used. The models were set up using a one-by-one backward removal of non-significant fixed effects (the criterion was 1.65 for a Wald test, one sided 0.05 significance). Next, one-by-one non-significant covariances were removed using a deviance test. The last step consisted of the removal (once more) of non-significant fixed effects. We followed this procedure to ensure we did not overlook suppression effects and that the covariance matrix was, initially, as free as possible. For the analysis the package MLwiN (version 1.10.000.6) was used (www.bristol.ac.uk/cmm/software/mlwin/). For details of the analysis see [45], [46].

For the clusters psychomotor performance, memory, and executive functions, four models were evaluated:

Model 0:  this model was used for reference.

Model 1:  group.

Model 2:  pain duration.

Model 3:  group and pain duration.

Model 4:  group, pain duration and consecutive covariates (depression, sleep disturbances, use of opioid medication, and a history of alcohol abuse).

These models were not nested, thus comparison of the models using the standard deviance test was not appropriate. We used the Akaike's Information Criteria corrected for small samples (AICc) to evaluate the multiple regression models and select the “best” model for cognitive functions for each cluster at two levels (see Appendix S2).

For the multivariate multilevel analysis the test score variables for the clusters psychomotor performance, memory, and executive functions, were standardized (range between −2 and 2, mean zero). The predictor ‘group’ was a dichotomous variable (patients versus healthy controls) and the predictor ‘pain duration’ was a continuous variable with standardized values within the patients group (where the value for the control group is arbitrary and chosen to be 0 as it does not vary within that group). All covariates were dichotomous variables, except for the depression score, which was continuous and these scores were standardized in the multilevel analysis (range between −2 and 2, mean zero).

Effects (E) on the test scores of the neuropsychological tests were calculated and in order to compare the effects, effect sizes (ES) for the continuous and dichotomous variables were calculated (see Appendix S3).

For a comprehensive illustration of the calculation and interpretation of estimated effects and effect sizes of the predictors ‘group’ and ‘pain duration’ see Appendix S4.

## Results

### Demography

The study sample consisted of 16 patients with chronic pancreatitis pain and 16 healthy controls. Patients and healthy controls were matched according to age, gender, and years of education (Table 1). Mean duration of chronic pancreatitis pain was 6 years, 8 patients had a history of alcohol abuse and 8 patients used opioid medication to relieve their pain (Table 1).

### Psychomotor performance

For the cluster psychomotor performance, model 2 with the predictor ‘pain duration’ (period of pain in years) had a lower AICc value (366.8) compared to the AICc of model 1 (376.2) including the predictor ‘group’ (patient or healthy control) (Table 3). Therefore, ‘pain duration’ was a better predictor than the predictor ‘group’ for the observed test scores on psychomotor performance. In addition, model 4 had the lowest AICc value (325.9). This model, including the predictors ‘group’ and ‘pain duration’ together with consecutive covariates, gave the best explanation (i.e. fit) for the variance in the observed test scores for psychomotor performance (Table 3). Table 4 shows the significant effects of ‘group’ and ‘pain duration’ for the cluster psychomotor performance with model 1, 2, 3 and 4.

**Table 3:** Statistical outcomes of the multivariate analysis.

		Model 0 reference	Model 1 group	Model2 duration	Model 3 group and duration	Model 4 group and duration and covariates
Psychomotor	Fit	328.0	320.7	311.2	300.1	193.4
	(cases, parameters)	(184,21)	(184, 24)	(184, 24)	(184, 28)	(178, 48)
	AICc	375.7	376.2	366.8	366.6	325.9
Memory	Fit	507.1	504.4	480.9	480.9	439.9
	(cases, parameters)	(283, 35)	(283, 35)	(273, 39)	(273, 39)	(270, 49)
	AICc	587.3	584.6	572.3	572.3	560.2
Executive	Fit	444.9	438.0	425.7	412.6	362.2
	(cases, parameters)	(191, 22)	(191, 25)	(191, 24)	(191, 30)	(185, 38)
	AICc	494.9	495.9	480.9	484.2	458.5

Fits (with corresponding cases and parameters) and AICc values for the clusters psychomotor -, memory - and executive functions.

**Table 4:** Significant effects of the multivariate analysis.

Cluster	Test nr	Variable measured	Model 1 group only	Model 2 duration only	Model 3 group and duration	Model 4 group and duration and covariates
			Estimate (SE)	Estimate (SE)	Estimate (SE)	Estimate (SE)^covariates^
**Predictor Group**						
Psychomotor	1	Tapping freq.(dominant)				−0.73 (0.27)^2^
		Tapping freq.(non-dominant)				−0.71 (0.28)^2,4^
		Tapping variability (dominant)			−0.43 (0.14)	−1.13 (0.26)^3,4^
		Tapping variability (non-dominant)				−1.49 (0.29)^1,3,4^
	2	Target detection (time)	−0.49 (0.26)		−0.44 (0.26)	−1.85 (0.34)^4^
	3	Choice Reaction Time	−0.44 (0.20)		−0.39 (0.20)	−0.51 (0.28)^1,4^
	4	Working Memory Reaction Time	−0.46 (0.25)		−0.48 (0.26)	−1.39 (0.38)^4^
Memory	5	Verbal word Learning trials (#)				^3,4^
		Verbal word Learning trials (slope)				
		Verbal word Delayed recall				0.49 (0.15)^4^
		Verbal word Recognition				−0.67 (0.31)^3^
	6	Maze A				0.55 (0.27)^1,3,4^
		Maze B				−0.59 (0.28)^1,4^
		Digit span forward task				
	7	Digit span backward task				
	8	Visual Span	0.29 (0.16)			^2^
Executive	9	Switching of Attention 1				−0.80 (0.22)
	10	Switching of Attention 2				
		Switching of Attention 2 (errors)			0.69 (0.22)	
	11	Verbal Interference (correct)	−0.60 (0.21)			−1.06 (0.14)^2,3^
		Verbal Interference (errors)				
	12	Intrusions			0.64 (0.25)	0.58 (0.27)^3^
	13	Go-NoGo	0.63 (0.36)		0.79 (0.33)	1.63 (0.42)^1^
**Predictor Pain duration**						
Psychomotor	1	Tapping freq.(dominant)		−0.55 (0.20)	−0.21 (0.06)	−0.62 (0.19)^2^
		Tapping freq.(non dominant)		−0.39 (0.22)		−0.47 (0.21)^2,4^
		Tapping variability (dominant)		−0.69 (0.20)	−0.40 (0.10)	−0.84 (0.16)^3,4^
		Tapping variability (non dominant)		−0.37 (0.22)		−0.75 (0.18)^1,3,4^
	2	Target detection (time)		−0.41 (0.20)	−0.34 (0.19)	−0.55 (0.17)^4^
	3	Choice Reaction Time				
	4	Working Memory Reaction Time				
Memory	5	Verbal word Learning trials (#)		−0.45 (0.24)	−0.45 (0.24)	^3^
		Verbal word Learning trials (slope)				
		Verbal word Delayed recall		−0.46 (0.22)	−0.46 (0.22)	
		Verbal word Recognition				^3^
	6	Maze A		−0.49 (0.24)	−0.49 (0.24)	−0.05 (0.01)^1^
		Maze B		−0.45 (0.24)	−0.45 (0.24)	
		Digit Forward task		−0.51 (0.24)	−0.51 (0.24)	−0.29 (0.14)
	7	Digit Backward task				
	8	Visual Span				^2^
Executive	9	Switching of attention 1		−0.62 (0.15)	−0.64 (0.15)	−0.49 (0.10)^2,3^
	10	Switching of attention 2		−0.35 (0.15)	−0.34 (0.15)	
		Switching of attention 2 (errors)		0.78 (0.18)	0.81 (0.17)	0.57 (0.14)^1,4^
	11	Verbal Interference (correct)				
		Verbal Interference (errors)				
	12	Intrusions				
	13	Go-NoGo				

*Estimates* and *SE* (standard error) from the multivariate multilevel analysis explaining test scores with predictors ‘group’ (upper panel) and ‘pain duration’ (lower panel). (*n* = 32, 16 pain patients).

Shown are the significant effects of predictors ‘group’ and ‘pain duration’ on test scores in the psychomotor -, memory - and executive functioning clusters with covariates of relevance.

All effects with *p*≤0.05.

Covariates (^1^ = depression, ^2^ = sleep disturbance, ^3^ = opioid medication, and ^4^ = alcoholism).

The effects of ‘group’ were significant for all seven tests when ‘pain duration’ and covariates were included (i.e. model 4), meaning that chronic pain patients performed worse on all tasks within the psychomotor cluster compared to the healthy controls (Table 2, Table 4 upper panel). Vice versa, the effect of ‘pain duration’ remained a significant predictor, when ‘group’ and covariates were included (model 4, Table 4 lower panel). In more detail, a comparison between the predictor ‘group’ and the predictor ‘pain duration’ on e.g. the ‘Tapping frequency’ tasks regarding effects showed that the effect sizes of ‘pain duration’ with significant estimated effects (−0.62, dominant hand) and (−0.47, non-dominant hand) were higher than the effect sizes of ‘group’ with significant estimated effects (−0.73 and −0.71 respectively) (Table 4). Thus on the ‘Tapping frequency’ tasks pain patients tapped significantly more slowly compared to healthy controls. In addition, tapping frequency was significantly further decreased in patients with long pain duration compared to patients with short pain durations. Also, the effect sizes for ‘pain duration’ with significant estimated effects (−0.84) and (−0.75) on the ‘Tapping variability’ of the dominant and non-dominant hand were higher than the effect sizes for ‘group’ with significant estimated effects (−1.13) and (−1.49). Patients showed significantly more variability between tapping compared to the healthy controls on this task and this variability significantly further increased with longer pain durations (Table 4). Similarly, in the ‘Target detection’ task the effect size of ‘pain duration’ with a significant estimated effect (−0.55) was stronger than the effect size for ‘group’ with significant estimated effect (−1.85) on the reaction times in this task. Thus, patients showed an increase in reaction time compared to the healthy controls and long pain durations were accompanied by prolonged reaction times compared to short pain durations in this ‘Target detection task’.

With respect to the domain of psychomotor performance which comprised seven tests, the covariate depression appeared to hold additional explanatory effects with respect to two tests: the “Variability’ in taps of the non-dominant hand and performance on the ‘Choice Reaction Time’ test. Similarly, a presence of sleep disturbance holds additional explanatory effects for two tests: ‘Tapping’ with the dominant hand and with the non-dominant hand. Finally, the use of opioid medication also holds additional explanatory effects for two tests: ‘Variability’ in taps of the dominant and the non-dominant hand. A history of alcohol abuse appeared to have additional explanatory effects with respect to the six tests: ‘Tapping’ with the non dominant hand, ‘Variability’ in taps of both the dominant and the non-dominant hand, ‘Choice Reaction Time’ test, ‘Target Detection’ test, and ‘Working Memory’ test. Alcohol abuse is therefore the covariate with the greatest impact.

### Memory

In the cluster memory, the ranking of the AICc values of the 4 different models showed that model 2 with the predictor ‘pain duration’ (AICc 572.3) was the better statistical model for explaining memory compared to model 1 with the predictor ‘group’ (AICc 584.6). Thus the predictor ‘pain duration’ was a better predictor than the predictor ‘group’ (chronic pancreatitis patient or healthy control) with respect to memory. However, this advantage disappeared when covariates were added in the model (Table 4). Model 4 had the lowest AICc value (560.2), so the predictors ‘group’ and ‘pain duration’ and consecutive covariates together best explained the observed test scores for memory performance (Table 3).

The estimated effects of ‘group’ in model 4 were significant for both parts of the ‘Maze learning’ tasks (0.55) and (−0.59), ‘Delayed recall’ of the verbal word learning task (0.49) and ‘Recognition’ of the verbal word learning task (−0.67) tests. Thus pain patients scored less accurately on these tasks compared to healthy controls (Table 2, Table 4 upper panel). In the test ‘Digits Forward’ task, long pain duration gave significantly decreased scores compared to short pain duration, with an estimated effect of (−0.29) for pain duration.

The cluster memory compromised nine tests. Depression appeared to be of relevance by having additional explanatory effects with respect to two tests (‘Maze learning’ A and B). In addition sleep disturbance had additional explanatory effects in one test: the ‘Visual span’ test. The use of opioid medication had additional explanatory effects with respect to three tests: ‘Maze learning A’, verbal word learning and recognition of verbal word learning. A history of alcohol abuse had additional explanatory effects in four tests within the cluster memory: maze learning A and B, verbal word learning and the delayed recall of verbal word learning.

### Executive functions

In the cluster executive functions, the model with ‘pain duration’ (AICc 480.9) better explained the test scores of executive functions than the model with ‘group’ (AIC 495.9). Multilevel analysis showed that duration of pain remained of importance when the covariates were included (model 4), according to the estimated effects (Table 4). Again, model 4 had the lowest AICc value (458.5) and therefore is the best fit for the test scores in the cluster executive functions (Table 3).

The estimated effects of ‘group’ were significant with respect to the tests ‘Switching of attention 1’ (−0.80), correct responses on the ‘Verbal interference’ test (−1.06), ‘Intrusions’ on the verbal word learning task (0.58), and reaction times to the ‘Go-NoGo’ task (1.63), meaning that patients showed a significant decline in executive functions compared to the healthy controls (Table 2, Table 4 upper panel). The predictor ‘pain duration’ had significant estimated effects for the speed in ‘Switching of attention 1’ test (−0.49) and errors on the ‘Switching of attention 2’ test (0.57). The effect size of ‘pain duration’ with significant estimated effect (−0.49) is higher compared to the effect size of group with significant estimated effect (−0.80) for the ‘Speed in switching of attention 1’ test. Depression had relevant explanatory effects next to ‘pain duration’ and ‘group’ with respect to two executive function tests: the number of errors in the ‘Switching of attention 2’ and the reaction times in the ‘Go-NoGo’ task. In addition, sleep disturbance had explanatory effects with respect to two tests: the speed in ‘Switching of attention 2’ (time) and on correct responses in the ‘Verbal interference’ test. The use of opioid medication had explanatory effects in three tests: correct responses on the ‘Verbal interference’ test, ‘Intrusions’ and speed of ‘Switching of attention 1’. Finally, a history of alcohol abuse only had additional effects on the errors on the ‘Switching of attention 2 test’.

**Figure 1:**
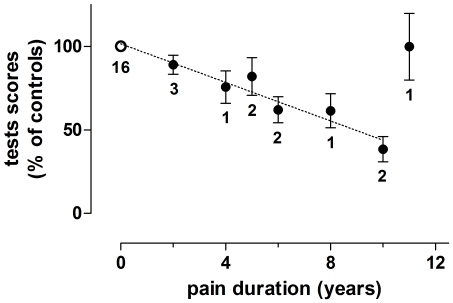
Pain duration dependent decrease in cognitive performance. The figure visualized the pain duration dependent decrease in cognitive performance. Test scores are depicted (ordinate) versus duration of pain in years (abscissa). Only the scores on those nine tests with p≤0.05, explaining test scores with predictors ‘pain duration’ are given (see Table 4 lower panel, model 4: ‘pain duration’). The mean of the scores of the controls (with pain duration zero) on each of the nine tests was taken as 100%. For those test where an increase in test score indicated a decrease in performance, the inverse of the raw scores was taken. The percentage of the test scores of each individual subjects was calculated. The mean and SE of all these percentages (so of all subjects on all nine tests) are shown. For each point the number of subjects is indicated. Remarkable is that the patient that had pain duration of 11 years had a mean test score on the nine tests comparable to the controls. This patient was a young patient of only 29 years old.

In summary, for each cognitive cluster, the predictor ‘pain duration’ (model 2) gave a better explanation for the variance in cognitive performance (i.e. had a lower AICc value) than the predictor ‘group’ (model 1) did. However, variance in all three cognitive domains could be best explained with model 4 which included ‘group’ and ‘pain duration’ together with relevant covariates of depression, sleep disturbance, opioid medication, and a history of alcohol abuse. The mean performance of the patients, in those nine tests that were significant deviant from controls with respect of pain duration, was 73% of that of the controls (SEM 3.4, n = 108 test values, Table 4 lower panel, model 4). The impact of the duration of the pain on this decline is visualized in figure 1.

## Discussion

The current study investigates whether chronic pancreatitis pain is accompanied by a decline in cognitive performance, and whether this decline could be related to neurodegenerative properties of the chronic pain. Neuropsychological profiles of patients suffering from chronic pancreatitis pain were compared to those from healthy matched controls.

We found that patients with chronic pancreatitis pain performed significantly worse on tests within all three cognitive domains compared to matched healthy controls. Moreover, the test scores could best be explained when pain duration was included as a second predictor, additional to being a patient or healthy control. Thus, longer pain durations were associated with greater declines in cognitive performance of patients and ‘pain duration’ resulted in larger effect sizes for predicting the test scores on cognitive tasks than ‘group’ did.

Pain duration particularly affects functions in the cluster psychomotor performance. Psychomotor performance strongly relies on the intactness of the frontal lobes. Thus the psychomotor slowing observed in the pancreatitis pain patients may be attributable to alterations of motor- and premotor cortices as well as midbrain structures regulating the general level of arousal (e.g. the thalamus) [47].

The findings of the current study related to the domain of psychomotor performance may further have been affected by all four covariates investigated. These factors have all previously been associated with a decrease in psychomotor speed [36], [38], [48], [49]. In the current study these four factors all offered some additional explanation for the observed decrease in psychomotor performance in the patients, but this appears less substantial than the explanatory effect of pain duration.

Pancreatitis pain patients also showed impairments in executive functions. Significant effects of pain duration were found on tasks that highly depended on mental flexibility (i.e. switching of attention task), self-monitoring abilities (i.e intrusions on word learning) and withholding a response (i.e. verbal interference). Executive functions represent a high, more abstract level of processing, and are mainly supported by the prefrontal cortex [50]. Interestingly, Apkarian et al., [29] observed a loss of cortical grey matter in patients suffering from chronic pain, especially in the frontal cortices and thalamus. In a subsequent study, a link between decreased grey matter in the prefrontal lobe and a reduced performance on an emotional decision-making task was suggested [26], [33]. The observed decline in psychomotor and executive performance observed in our pancreatitis pain patients might thus also be, at least partly, ascribed to a loss of grey matter in the frontal cortices and thalamus.

Memory performance was the least affected cognitive function in patients with chronic pancreatitis. Mild problems with memory functioning have previously been related to depressive symptoms [35], [36], [51]. Thus, a mild decline in memory might be related to the increased depression scores found in the patients compared to healthy controls. Although previous studies of patients with chronic pain often have reported memory deficits [22], [52], this domain is only mildly affected within the current study.

Depression, sleep disturbance, use of opioids, and a history of alcohol abuse, are all factors that have been associated with decreased cognitive abilities. Therefore, in the current study these factors were included in the models explaining the observed variance in neuropsychological test data. Indeed, with respect to a number of the tests, these factors did offer additional explanation for the observed cognitive decline in pancreatitis patients. Of these factors, a history of alcohol abuse appeared to be the most prominent factor. However, the effect sizes of a history of alcohol abuse were still modest in comparison with the effect sizes of chronic pain and pain duration (data not shown). This limited effect might be ascribed to the long duration of alcohol abstinence at time of testing in our patients, i.e. at least one year. Indeed, significant recovery has been found within one year of abstinence in most cognitive domains [53], [54], [55], [56]. Fein et al. examined cognitive performance in long-term abstinent middle-aged alcoholics and found that abstinent alcoholics performed similarly to controls in all areas of cognitive performance, except for a minor deficit in spatial processing [55].

In this study a homogenous group of patients was recruited, all having a confirmed diagnosis of pancreatitis. Despite the patients being homogenous in the cause of the pain, it is still difficult to ascribe the observed cognitive deficits to just one underlying cause. This difficulty is not only due to the variation in the duration of their pain disease but also due to comorbidity with depression, sleep disturbances, the high prevalence of a history of alcohol abuse and a current use of opioid medication. However, by applying multivariate multilevel analyses we were able to entangle at least partially the differential influences of these contributing factors.

The uniqueness of this study is that it is the first study to formally assess the cognitive performance of chronic pancreatitis pain patients. Previous studies in this field have focused on other chronic pain patients e.g. low back pain [57] and fibromyalgia [23], [25], [58], or mixed pain conditions in patients with chronic non-malignant pain [22], [59]. The similar findings of a decline in cognitive performance in these previous studies and those in the present study support the concept that the chronic pain itself is the denominator of cognitive decline and not the associated pathology giving rise to the chronic pain syndrome.

The direct detrimental effect of pain duration on cognitive performance in the present study is a new observation, which has not been reported previously. This negative effect of pain duration on cognitive performance supports the novel concept of viewing chronic pain as a disease with neurodegenerative properties. From a therapeutic perspective, the suggestion that neurodegeneration may be related to chronic pancreatitis pain is extremely relevant. Typically, chronic pancreatitis patients with pain are treated with pain medication including opioids over long periods of time, with limited treatment success and low health-related quality of life, predominantly as a result of persisting or relapsing pain despite medication [60]. As a consequence, patients frequently become unemployed, and may even be deprived of the ability to indulge in social and sport activities [8]. In this context, earlier and more effective therapeutic interventions targeting not only the cause of pain or blocking sensory input, but also specifically addressing the associated central neuroplasticity might reduce or prevent neurodegeneration and decline in cognitive performance, thereby improving the pain outcomes and quality of life in these patients.

## Supporting Information

Appendix S1 **Appendix S1 gives a detailed description of the neuropsychological tests used.**
(DOCX)

Appendix S2 **Appendix S2 shows the Akaike's Information Criteria.**
(DOCX)

Appendix S3 **Appendix S3 shows how effects were calculated.**
(DOCX)

Appendix S4 **Appendix S4 shows a sample calculation of effects and effect sizes of the predictors.**
(DOCX)
