# Supplementary material for: Neurodegenerative Properties of Chronic Pain: Cognitive Decline in Patients with Chronic Pancreatitis
Source: PLoS One. 2011 Aug 18;6(8):e23363. doi: 10.1371/journal.pone.0023363 (PMC3158076; doi:10.1371/journal.pone.0023363)
Supplement: Appendix S1 — gives a detailed description of the neuropsychological tests used. (DOCX) [file pone.0023363.s001.docx]

Appendix S1

*Psychomotor functions*

1. The Motor Tapping test is a variation of the Finger Tapping test that provides an index of manual dexterity. The participant was required to tap for 30 sec. with the index finger as fast as possible on a touch screen. Measured were the total number of tabs and the variability of the time between taps. Data for both the dominant hand and the non-dominant hand were determined.

2. Within a target detection task, target and background tones were presented in a quasi-random order, such that two target tones did not appear consecutively. Participants were instructed to button press with the index finger of each hand (to counterbalance for possible motor effects) in response to target stimuli. Measured was the average reaction time.

3. With the Choice Reaction Time test, four black circles were presented equally spaced along a semicircular arc across the top of the touch screen. Periodically and with equal probability, a black circle was illuminated in green. The participant was required to touch each illuminated circle as fast as they could. The average reaction time was measured.

4. The Working Memory task consisted of a series of letters (B, C, D, or G) presented to the participant on the touch screen. Participants were instructed to press a button with both index fingers simultaneously if the same letter appeared twice in a row. The dependent variable reported is reaction time.

*Memory functions*

5. Memory and learning were tested using a verbal recall task called Verbal List-Learning in which the participant was presented with a list of 12 words via a headset. Participants were required to recall as many words as possible. This procedure was repeated 4 times (learning trials 1-4). Participants were then presented with a list of distracter words and asked to recall them after presentation. Following this, they were asked to recall the 12 words from the original list. After about 25 minutes they were asked to recall again the 12 words from the original list. The participant was then presented one at a time with a series of 24 words on the computer screen (Recognition trial). Half of these words were the words from the first list; the remaining words were new words. Following each word, the participant was required to touch a “Yes” or “No” button on the touch screen according to whether or not the word was in the original list. Measured were the number of learned words in the four trials and the slope of the learning curve.

6. The Maze task was used to assess planning, foresight, and self-monitoring during the course of learning and remembering a complex maze. The maze contained a hidden path and it was the participant’s task to discover and remember this path. Measured was the time to finish the task.

7. The Digit Span Forward task provides a verbal measure of short-term memory. In this task, the participant had to repeat a list of digits in the same order as auditory-presented sequences. As a measure of working memory functions, we used the Digit Span-Reversed task, in which the participant had to repeat a list of digits in the reversed order. The score is the longest sequence length correctly completed.

8. Similarly, within the Visual Span task a sequence of squares was presented at different positions on a touch screen. The participants had to reproduce these sequences. This task was used to provide an index of visual short-term memory capacity. The dependent measure is the longest sequence length correctly completed.

*Executive functions*

9. Part 1 of the Switching of Attention test is an adaptation of the Trail Making test [[1](#_ENREF_1)] and addressed visuomotor tracking and motor speed. The participant was presented with a display of 25 numbers scattered at random on the computer screen and asked to touch the numbers in ascending numerical sequence (i.e. 1 2 3 . . .). The dependent variable reported is the time taken to complete the test successfully.

10. In part 2 of the Switching of Attention task, the participant was presented with a random pattern of 13 numbers (1–13) and 12 letters (A–L) on the screen and was required to touch numbers and letters in both an ascending and alternating manner (i.e. 1 A 2 B 3 C . . .). This part is more difficult than the first part and reflects the requirement to switch attention between two stimulus sets and thereby alternate between the respective mental sets involved. The dependent variable reported is the time to completion.

11. The Verbal Interference test is an adaptation of the Stroop test. This task consisted of a series of colored words presented one at a time on the computer screen. The color and name of each colored word was always one of four colors (blue, green, yellow and red) with the constraint that no colored word had the same color and name. In part 1 of this test, the participant was required to identify the name of each colored word as quickly as possible after it was presented on the screen. Part 2 of the Verbal Interference test measured the ability to suppress the automatic well-learned responses of reading. In this part, the participant was required to name the color of each colored word as quickly as possible after it was presented on the computer screen, but ignore the written meaning of the word. The dependent measures obtained were the number of words correctly identified in part 1 and the number of colors named incorrectly in part 2.

12. From the Verbal List-Learning, the number of intrusions (words incorrectly recalled from the distracter list on the short delay recall trial) was scored. The measure of executive functioning obtained from the Verbal Memory Recall task are the number of words incorrectly recalled during trials 1–4 (Intrusions).

13. Finally, in a Go-NoGo task, participants were presented with repetitions of the word “PRESS” on the screen in front of them. Participants were instructed to press (once) a button with the index finger of each hand whenever they saw the word presented in green font and refrain from pressing the response buttons, whenever the word appeared in red font. Measured was the averaged reaction time.

1. Reitan RM (1955) The relation of the trail making test to organic brain damage. Journal of consulting psychology 19: 393-394.
