# Supplementary material for: Neurodegenerative Properties of Chronic Pain: Cognitive Decline in Patients with Chronic Pancreatitis
Source: PLoS One. 2011 Aug 18;6(8):e23363. doi: 10.1371/journal.pone.0023363 (PMC3158076; doi:10.1371/journal.pone.0023363)
Supplement: Appendix S2 — shows the Akaike's Information Criteria. (DOCX) [file pone.0023363.s002.docx]

Appendix S2

The best fitting model can be defined as the one with the lowest expected information loss and is asymptotically equivalent to choosing a model *M*_i_, i = 1, 2, …, *K* with the lowest AIC value [[1](#_ENREF_1),[2](#_ENREF_2)].

The AIC is defined as:

 (1)

“Where *L_i_*, the maximum likelihood for the candidate model *i,* is determined by adjusting the *V_i_* free parameters in such a way as to maximize the probability that the candidate model has generated the observed data” [[3](#_ENREF_3)]. In this study the finite sample correction is used, which is recommended when *n* < 40 [[4](#_ENREF_4)].

The AIC_c_ is defined as:

$\frac{2V\left(V+1\right)}{\left(n-V-1\right)}$ (2)

1. Akaike H. Information theory and an extension of the maximum likelihood principle. In: Petrov BN, editor; 1973; Budapest, Akademiai Kiado. pp. 267-281.

2. Bozdogan H (1987) Model selection and Akaike’s information criterion (AIC): the general theory and its analytical extensions. Psychometrika 52: 345-370.

3. Wagenmakers EJ, Farrell S (2004) AIC model selection using Akaike weights. Psychonomic bulletin & review 11: 192-196.

4. Burnham KPA, D.R. (2002) Model selection and multimodel inference: a practical information-theoretic approach New-York: Springer-Verlag.
