# Supplementary material for: Neurodegenerative Properties of Chronic Pain: Cognitive Decline in Patients with Chronic Pancreatitis
Source: PLoS One. 2011 Aug 18;6(8):e23363. doi: 10.1371/journal.pone.0023363 (PMC3158076; doi:10.1371/journal.pone.0023363)
Supplement: Appendix S3 — shows how effects were calculated. (DOCX) [file pone.0023363.s003.docx]

Appendix S3

An effect size (ES) is defined as:

$ES=$ $\frac{E*R_{p}}{R_{y}}$ (3)

$R_{p}$: Range of predictor

$R_{y}$ : Range of dependent variable

Because all continuous variables were standardized the $R_{p}$and $R_{y}$ have the value 4 (mean +/- 2 standard deviation; standard deviation = 1). For the dichotomies variables the range is 1 (possible values 0 and 1).

An effect size of continuous variable is defined as:

$ES_{c}=E$ (4)

Thus, an effect size of dichotomous variable is defined as:

$ES_{d}=\frac{E}{4}$ (5)
