# Supplementary material for: Neurodegenerative Properties of Chronic Pain: Cognitive Decline in Patients with Chronic Pancreatitis
Source: PLoS One. 2011 Aug 18;6(8):e23363. doi: 10.1371/journal.pone.0023363 (PMC3158076; doi:10.1371/journal.pone.0023363)
Supplement: Appendix S4 — shows a sample calculation of effects and effect sizes of the predictors. (DOCX) [file pone.0023363.s004.docx]

Appendix S4

To illustrate the impression of both the effects (E) and effect sizes (ES) of the predictors group and pain duration on the test score the first test of the tapping task (Tapping dominant) will be explained.

The effect for group for the test Tapping dominant is $\left(-0.73\right)$ (Table 4a).

The effect size of group for the Tapping dominant test is:

$ES_{d}=\frac{-0.73}{4}=-0.18$ (6)

This means the control group $\left(-0.73*0=0\right)$ performs better on the Tapping dominant test compared to the patient group $\left(-0.73*1=-0.73\right)$. This range $\left(0.73\right)$ is 18% of the total test score range $\left(-2...+2=4\right)$ of the cognitive test Tapping dominant.

The effect of pain duration for the test Tapping dominant is $\left(-0.62\right)$ (Table 4b).

Thus, the effect size of pain duration for the Tapping dominant test is:

$ES_{c}=-0.62$ (7)

This means low pain duration $\left(-0.62*-2=1.24\right)$ will lead to a better cognitive functioning on this test compared to high pain duration $\left(-0.62*2=-1.24\right)$. This range $\left(1.24*2=2.48\right)$ is $62\%$ of the total test score range $\left(-2...+2=4\right)$ of the psychomotor test Tapping dominant.
